# 

**Published:** 1869-08-15

**Authors:** 


Vol. XXVI. Numbers 15 and 16.

THE

### CHICAGO

### Medical Journal.

PUBLISHED SEMI-MONTHLY.

J. ADAMS ALLEN, M. D., LL. D.,

WALTER HAY, A. M., M. D.,

*EDITORS.*

**AUGUST 1st and 15th.**

1869.

J. WOLCOTT HOOKER, 12 Union Building,

CHICAGO.

HORTON & LEONARD, Printers, 104 and 106 Randolph Street.

